# Iron supplementation during pregnancy – a cross-sectional study undertaken in four German states

**DOI:** 10.1186/s12884-018-2130-5

**Published:** 2018-12-13

**Authors:** Irmela Rosina Demuth, Annett Martin, Anke Weissenborn

**Affiliations:** 1 Department of Food Safety, German Federal Institute for Risk Assessment, Max-Dohrn-Str. 8-10, 10589 Berlin, Germany; 2 Current Address: Department of Nutritional Behaviour, Federal Research Institute of Nutrition and Food (Max Rubner-Institut), Haid-und-Neu-Straße 9, 76131 Karlsruhe, Germany

**Keywords:** Iron, Pregnancy, Supplementation, Anaemia, Iron deficiency, Iron overload

## Abstract

**Background:**

Iron deficiency but also iron overload during pregnancy has been associated with unwanted health effects. In Germany, iron supplements are only recommended for pregnant women with diagnosed iron deficiency/anaemia. Prevalence of anaemia among pregnant women was reported at 24.4% in 2011. However, limited data suggest that more than 60% of women in Germany use iron supplements during gestation. Against this background, we investigated the prevalence of iron supplement intake among pregnant women and explored determining factors in order to assess whether women are following the advice to only supplement iron in case of a diagnosed iron deficiency/anaemia.

**Methods:**

A cross-sectional study was carried out in four German states in 2015 where, with the help of midwives, women in childbed were asked to retrospectively answer a questionnaire about iron intake from various sources and reasons for supplementing iron during their recent pregnancy. We used Chi-square-tests and logistic regression analysis to evaluate associations between iron supplementation and other nutritional, sociodemographic and maternal variables and to assess attitudes of women meeting versus not meeting the official recommendation on iron supplement intake during pregnancy.

**Results:**

Of 207 participants, 65.2% had supplemented iron. 84.4% reported to have done this because of a diagnosed iron deficiency/anaemia. Iron intake ranged from 5 to 200 mg/day, and duration of supplementation varied between two weeks and throughout gestation. Of women who reported to have been diagnosed with iron deficiency/anaemia, 47.5% had supplemented ≥80 mg/day iron, while 26.2% had taken iron in lower amounts ≤40 mg/day. Six percent of the participating women had not supplemented iron in spite of a diagnosed iron deficiency/anaemia, whereas 19.7% of women without iron deficiency/anaemia still had supplemented iron (range: 7 to 80 mg/day).

**Conclusion:**

The majority of pregnant women used iron supplements in case of a diagnosed iron deficiency/anaemia. However, not all women with iron deficiency/anaemia supplemented (sufficient amounts of) iron, while there was also indiscriminate use of iron supplements in women without iron deficiency/anaemia. Further research is warranted to confirm these findings in representative samples.

**Electronic supplementary material:**

The online version of this article (10.1186/s12884-018-2130-5) contains supplementary material, which is available to authorized users.

## Background

During pregnancy, a balanced diet with an adequate intake of essential nutrients is important for foetal development and birth outcome, but also for the mother’s health. One of the micronutrients of special importance is iron. Being a vital constituent of haemoglobin, iron is essential for blood formation and oxygen supply, and it enables various enzymatic reactions in the human body. During gestation, iron requirements increase, most importantly due to an increase in the red blood cell mass and growth of the unborn child and placenta and mainly during the second and third trimester [1–3]. The total quantity of iron required for a singleton pregnancy is estimated to be 835 mg [4]. To provide for this, the Nutrition Societies of Germany, Austria and Switzerland (D-A-CH) recommend a daily iron intake of 30 mg for pregnant women from the second trimester of pregnancy. This amount is twice as high as the dietary reference value (DRV) of iron set for the general female non-pregnant population [5]. The European Food Safety Authority (EFSA), on the other hand, considered that no additional iron is required in pregnancy because menstruation ceases and iron absorption increases significantly during that time, and thus derived for pregnant women a DRV equal to that for non-pregnant premenopausal women of 16 mg/day [4].

According to the latest National Food Consumption Survey (NVS II) conducted in Germany in 2005–2007, non-pregnant women of reproductive age achieved a median daily iron intake of approximately 11–12 mg through normal diet [6]. These data suggest that more than 50% of women of that age neither achieve the DRV set by D-A-CH and EFSA for non-pregnant women of 15 and 16 mg/day, respectively, nor the much higher reference intake value of 30 mg/day set by D-A-CH for pregnant women in Germany [4, 5]. Women in Germany are thus at high risk for inadequate pre-pregnancy iron stores and for developing iron deficiency (ID) or iron deficiency anaemia (IDA) during gestation.

Maternal anaemia during pregnancy, especially in the second trimester, influences postnatal infant growth [7] and is associated with an increased risk for low birth weight and preterm birth [8, 9]. This emphasizes the importance of an adequate iron status during gestation. Yet, routine iron supplementation in non-anaemic, well-nourished pregnant women increases the risk for developing high levels of haemoglobin in the late pregnancy or postpartum period [10] and may result in significant increase of reactive oxygen species [11] and lipid peroxidation [12]. Further, evidence suggests that the intake of (high-dosed) iron supplements in iron-replete pregnant women or in women with elevated iron stores may also be associated with negative effects such as low birth weight, preterm birth and an increased risk of gestational diabetes [9, 13–17]. Altogether, evidence supports the effectiveness of iron supplementation for improving maternal haematological parameters, although the clinical significance for both pregnant women and infants remains unclear [10, 18]. In view of that, the US Preventive Services Task Force (USPSTF) concluded that there is currently insufficient data to assess the balance of benefits and harms of routine iron supplementation for the health of pregnant women and their birth outcome [18]. International guidelines and recommendations on iron supplementation during pregnancy vary, however: While some countries (e.g. Canada, USA) recommend routine iron supplementation with daily doses of around 30 mg of iron, others (e.g. UK, France, Ireland) only recommend iron supplements in case of symptoms of ID/IDA [19–21]. Taking into consideration that anaemia is one of the major public health problems that affects mainly low- and middle-, but also high-income countries, and has significant adverse health consequences as well as adverse impacts on social and economic development, reducing anaemia is recognized as an important component of the health of women and children, and thus, World Health Organization (WHO) recommends universal iron supplementation of pregnant women [22].

In Germany, medical societies recommend iron supplementation in pregnancy only for women with a diagnosed anaemia [23]. Taking into consideration that the prevalence of anaemia among pregnant women in Germany is much lower than that reported from low- and middle-income countries (according to WHO [24], anaemia among pregnant women in Germany was 24.4% in 2011). Considering the ongoing discussion about risks associated with (high-dosed) iron supplements in iron-replete pregnant women, the recommendation given in Germany is considered prudent. There is, however, only limited information on how this is implemented into practice in Germany [25, 26].

Against this background and in order to assess whether the advice to only supplement iron in case of a diagnosed iron deficiency/anaemia is followed, the aim of this study was to investigate the prevalence and determining factors of iron supplementation in pregnant women in Germany and to examine onset, duration, iron dosage and compounds used for supplementation as well as other sources of iron intake.

## Methods

### Study design and participants

In order to gain insight into the iron supplementation practice and influencing factors in pregnancy, a cross-sectional study was carried out from July until mid-September 2015 in four German federal states (Berlin, Brandenburg, Lower Saxony and Hesse). The four states were chosen mainly for practical reasons such as access to midwives who contributed to the study by recruiting participants. However, the inclusion of more than one study site also meant that regional differences in counselling and supplementation practices could be explored.

All midwives (*n* = 52) who had agreed to support this study by approaching women in childbed were provided with a certain number of questionnaires, i.e. in average 15 per midwife. This number was determined on the basis of the estimated number of women for whom a midwife would have to care for within the specified study period of 2.5 months.

Women in childbed who were eligible for this study were randomly contacted by their midwife and provided with verbal and written information about the aim and purpose of the study. If interested in participation, they were requested to answer a written questionnaire about their most recent pregnancy. Completed questionnaires had to be sent back by the participants to the German Federal Institute for Risk Assessment where data were processed and analysed. Eligible for participation in this study were women with a minimum age of 18 years who had given birth to a child in the previous four weeks before answering the questionnaire and who had sufficient knowledge of German.

The study was fully anonymous as questionnaires were marked with code numbers only. Participants were provided with written information about the aim and purpose of the study and were offered a copy of that information for their records. The study was ethically approved without full board review by the Ethics Committee of the Berlin General Medical Council and the Ethics Committees of the three other federal states (Ethik-Kommission der Landesärztekammer Brandenburg, Ethik-Kommission der Ärztekammer Niedersachsen, Ethik-Kommission der Landesärztekammer Hessen). Also, due to the fact that the research design involved no risk and no identifiable private information, the request to obtain a signed consent from participants had been waived in conformity with the CIOMS International Ethics Guidelines [27].

### The questionnaire

The questionnaire was designed to determine information from participating women about iron supplement intake in their most recent pregnancy and reasons for supplementation. Furthermore, information about the nutritional intake^1^ of iron during pregnancy including the use of other dietary supplements and consumption of fortified foods was gathered in order to determine the contribution of other food sources to the overall iron supply. For further details, an English version of the questionnaire is included as Additional file 1.

To test the feasibility and comprehensibility of the questionnaire, a pre-test was performed beforehand and the questionnaire was adjusted accordingly. It took around 15 min to complete the questionnaire.

### Statistical analysis

All data were tested for plausibility and analysed with SPSS for Windows (PASW 18.0). Chi-square-tests (univariate analysis) and logistic regression analysis (multivariate analysis) were performed in order to evaluate associations between iron supplementation and other nutritional, sociodemographic and maternal variables. For this purpose, the study population was divided into women who had supplemented iron and those who had not supplemented iron during pregnancy. Furthermore those who had supplemented iron because of an ID/anaemia were divided from those who had done that due to other reasons. The population was also subdivided into women with a university degree (high education) and other graduates (lower education) and into two age groups: below and equal or above the median age, i.e. ≤ 32 and > 32 years.

The non-parametric Mann-Whitney U test was used to compare daily intake of iron supplements between women with and without ID/anaemia. Multivariate logistic regression analysis with a backward selection approach was used to identify the independent impact of each factor on iron supplementation. The level of significance was defined to be 0.05. Figures were created with Microsoft® Excel 2010 and RStudio (packages “ggplot2” and “yarrr”, https://www.r-project.org).

## Results

### Participants

In total, 52 midwives supported the study by contacting women in childbed during their regular postpartum visits and by providing study information and questionnaires to them. Of 782 questionnaires that had been sent out to these midwives, 209 were received back within the study period of two and a half months. Two questionnaires had to be excluded from analysis because data were not plausible. Thus, 207 questionnaires were analysed, resulting in a response rate of 26.5%. Participants were equally distributed among the four different federal states as follows: Berlin: *n* = 51 (24.6%); Brandenburg: *n* = 57 (27.5%); Lower Saxony: *n* = 53 (25.6%); Hesse: *n* = 43 (20.8%). In three cases (1.4%), the origin of the respondents was not known.

### Sociodemographic data

The mean age of the participating women was 31.8 years (median: 32 years). Most of them were of German origin (*n* = 185; 89.4%) and had a university degree (*n* = 121; 58.5%). About 55% of the women (*n* = 114) had given birth to their first child. In the majority of multiparous women, spacing between pregnancies was more than one year (*n* = 61; 66.3%). Five women (2.4%) reported that they had smoked and only one woman (0.5%) admitted to have consumed alcohol during pregnancy.

### Iron supplement intake

Of the 207 respondents, 65.7% (*n* = 136) indicated that they had supplemented iron during pregnancy. Nearly all women had had their iron status measured (*n* = 195; 94.2%) and, based on self-reported information from participants, two thirds of them (*n* = 121; 62.1%) had been diagnosed with ID/anaemia at some stage of pregnancy. The majority of cases had been detected in the second (*n* = 55; 45.5%) or third trimester (*n* = 40; 33.1%), and nearly one fifth (*n* = 22; 18.2%) already in the first trimester of pregnancy.

Women who had supplemented iron, reported to have done so mainly because of a diagnosed ID/anaemia (*n* = 115; 85.2%). However, (indiscriminate) iron supplementation was also reported by 15% (*n* = 21) of women who had either not been detected with ID/anaemia or could not report about it because their iron status had not been examined.

**Fig. 1:**
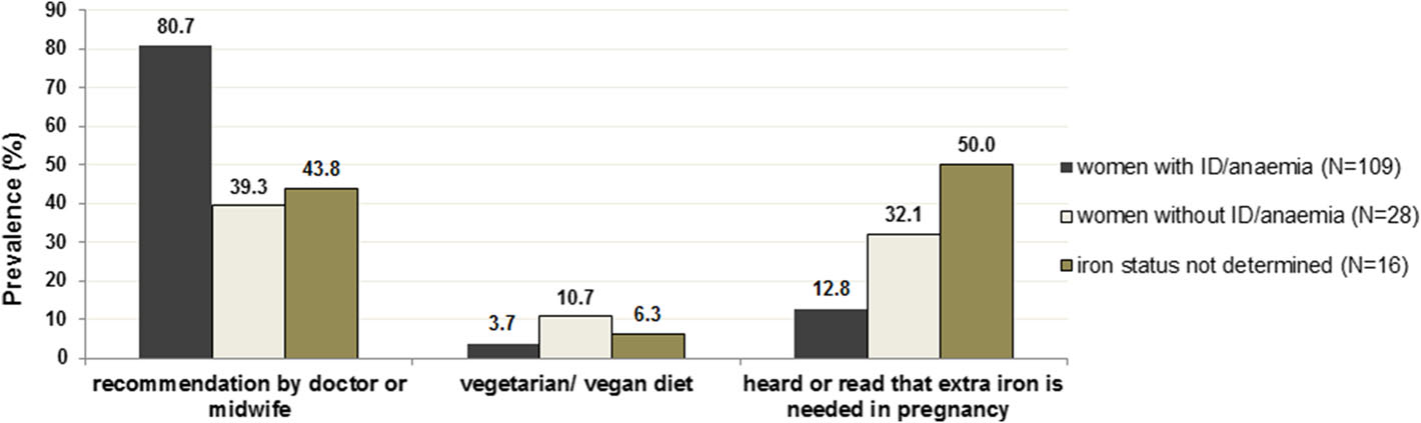
Reasons for iron supplementation during pregnancy

In most of the cases, women indicated that a doctor and/or midwife had advised them to use an iron supplement (*n* = 111; 77.9%). However, when looking separately at women with ID/anaemia (*n* = 109) and those who had not been diagnosed with (*n* = 28) or screened for (*n* = 16) ID/anaemia, 39.3% of those without ID/anaemia and 43.8% of women who’s iron status had not been determined, had also received a recommendation for iron supplementation from their doctor or midwife. Additional reasons for iron supplementation were a vegetarian diet, which had been practiced by 6.3% of the whole study group (*n* = 13), or that women had heard or read about benefits of iron supplement use during pregnancy (Fig. 1).

### Univariate analyses

Potential associations of sociodemographic, maternal or nutritional variables with iron supplementation were evaluated, and results are presented in Table 1. Univariate analyses revealed a highly significant association between ID/anaemia and the use of iron supplements (*p* <  0.001) whereas none of the other associations tested between sociodemographic factors and iron supplementation were significant. With respect to nutritional variables assessed, women who reported to be vegetarians (*p* = 0.04) as well as those who had taken other dietary supplements (*p* = 0.03) were more likely to have used iron supplements during pregnancy. Moreover, women who had consumed iron-fortified foods during pregnancy had a greater tendency to have used iron supplements, though without significance (*p* = 0.06) (Table 1).

**Table 1:** Associations between iron supplementation (n [%]) and sociodemographic/maternal data and determining factors of iron supplementation

	Users (*n* = 135)	Nonusers (*n* = 72)	Chi-square [OR (95%-CI)]	*P-value* ^1^	*Logistic regression* [OR (95%-CI)]	*P-value* ^2^
Sociodemographic/maternal data						
Home country (*n* = 200)						
Germany	118 (63.8)	67 (36.2)	0.44 (0.12; 1.62)	0.27		
Foreign country	12 (80.0)	3 (20.0)	1			
Age of women (*n* = 206)						
< 32 years	69 (71.1)	28 (28.9)	1			
≥ 32 years	66 (60.6)	43 (39.4)	0.62 (0.35; 1.12)	0.11		
Education (*n* = 207)						
Low education	56 (65.1)	30 (34.9)	1			
High education	79 (65.3)	42 (34.7)	1.01 (0.56; 1.80)	0.98		
Parity (*n* = 206)						
primipara	71 (62.3)	43 (37.7)	1			
multipara	63 (68.5)	29 (31.5)	1.32 (0.74; 2.35)	0.35		
Years in-between births (*n* = 80)						
More than one year	13 (68.4)	6 (31.6)	1			
Less than one year	43 (70.5)	18 (29.5)	1.10 (0.36; 3.35)	0.86		
Iron Status						
Iron deficiency (*n* = 187)						
Yes	114 (94.2)	7 (5.8)	50 (25.0; 166.7)	< 0.001	91.07 (28.99; 289.07)	< 0.001
No	13 (19.7)	53 (80.3)	1		1	
Time of ID/ anaemia diagnosis						
first trimester	22 (100.0)	0 (0.0)	1			
second trimester	53 (96.4)	2 (3.6)	1.04 (0.99; 1.09)	1.00		
third trimester	35 (87.5)	5 (12.5)	1.14 (1.02; 1.29)	0.15		
Diet/ life-style						
Intake of iron fortified food (*n* = 207)						
Yes	51 (73.9)	18 (26.1)	1.82 (0.96; 3.44)	0.06		
No	84 (60.9)	54 (39.1)	1			
Use of other food supplements (*n* = 206)						
Yes	108 (69.7)	47 (30.3)	2.04 (1.07; 3.90)	0.03	5.29 (1.52; 18.36)	0.02
No	27 (52.9)	24 (47.1)	1		1	
Dietary behaviour (*n* = 207)						
Mixed diet	123 (63.4)	71 (36.6)	1			
Vegetarian diet	12 (92.3)	1 (7.7)	6.93 (0.88; 54.39)	0.04*		
Alcohol consumption (*n* = 207)						
Yes	1 (100.0)	0 (0)				
No	134 (65.0)	72 (35.0)		1*		
Smoking (*n* = 204)						
Yes	3 (60.0)	2 (40.0)	1			
No	130 (65.3)	69 (34.7)	1.26 (0.20; 7.70)	1*		

*OR* odds ratio, *CI* confidence interval, ^1^ univariate analysis; ^2^ multivariate analysis; ^*^Fisher’s exact test

### Multivariate analysis

The multivariate analysis revealed that only two factors had an independent influence on the use of iron supplements during pregnancy, i.e. the prevalence of ID/anaemia (*p* < 0.001) and the use of other dietary supplements (*p* = 0.02) (Table 1).

### Dose, commencement, and duration of intake of iron supplement intake

**Fig. 2:**
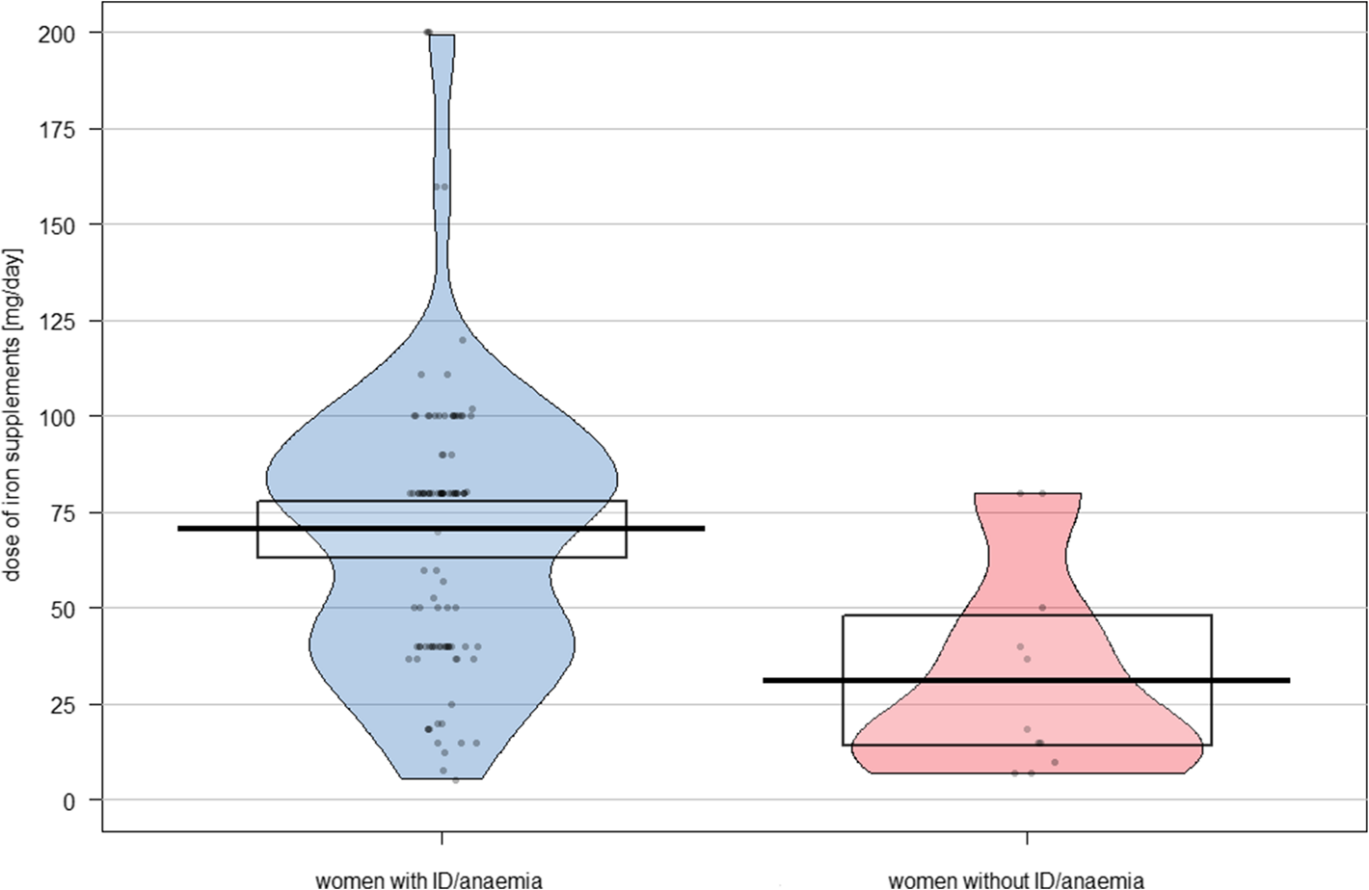
Daily dose of iron supplements [mg] of 115 women with and without ID/anaemia

Based on the respondents’ information, the daily dose of iron could be determined in 119 cases of supplement users (88.1%). According to these data, the overall median iron intake via supplements was 80 mg/day (mean: 65.5 mg/day). In women with ID/anaemia, intake ranged from 5 to 200 mg/day [median: 80 mg/day, mean: 70.5 mg/day (95% CI: 63.0–78.0 mg/day)] and in women without ID/anaemia from 7 to 80 mg/day [median: 16.7 mg/day, mean: 31.2 mg/day (95% CI: 14.3–48.1 mg/day)] (*p* < 0.001, U = 223). The mean daily intake of iron supplements with 95% confidence interval (CI) is shown in Fig. 2, which represents also all single values (the horizontal line represents the mean; the rectangle represents the 95% confidence interval; the shape of the figure represents a smoothed density).

**Fig. 3:**
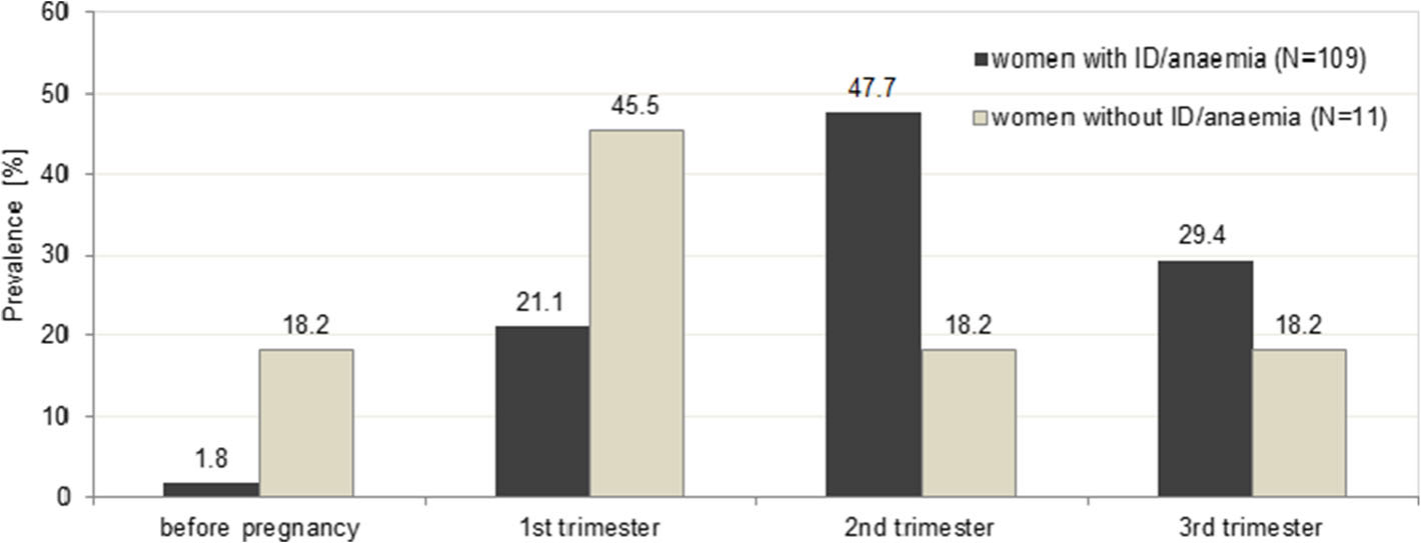
Onset of iron supplementation

The timing of commencement of iron supplementation differed widely, but nearly half of the women with ID/anaemia had started in the second trimester (*n* = 52; 47.7%) whereas most of the women without ID/anaemia reported to have started already in the first trimester or even before pregnancy (*p* = 0.02) (Fig. 3). With respect to the duration of iron supplementation, women with ID/anaemia did not supplement significantly longer than those who had reported other reasons for taking iron supplements (*p* = 0.15).

Products for which it was known (*n* = 114; 84.4%) contained iron compounds in a divalent form such as ferrous sulphate (*n* = 42; 36.8%) with a mean dose of 78 mg/day, ferrous glycine sulphate (n = 42; 36.8%) with a mean dose of 105 mg/day, or iron gluconate (*n* = 25; 21.9%) with a mean dose of 55 mg/day.

### Nutritional behaviour and other iron sources

**Fig. 4:**
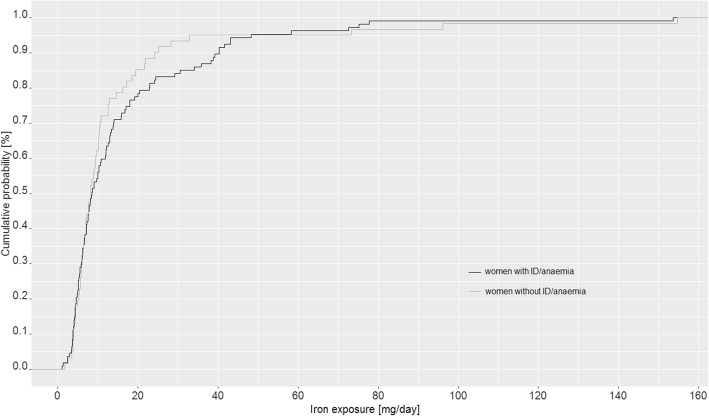
Nutritional iron intake, including fortified foods and dietary supplements of women with and without ID/anaemia

The majority of women (*n* = 190; 91.8%) provided information about their nutritional behaviour during pregnancy. According to this, three quarters of the study population had taken other dietary supplements (*n* = 155; 74.9%) in addition to iron supplements. Some of those products (*n* = 12; 7.7%) also contained iron with a median daily dose of 7.5 mg (range: 2–15 mg/day). Also, more than one third of the study population (*n* = 69; 36.3%) had consumed iron-fortified foods during pregnancy, of which fruit-based beverages were the most commonly reported (*n* = 60; 87.0%). The median contribution of fortified foods to iron intake was 10 mg/day. Altogether, the median iron intake of the study group estimated from various dietary sources (supplements, fortified foods, normal foods) was about 8 mg/day with no difference between women with and without ID/anaemia (*p* = 0.47). However, the 75th and 90th percentiles of women with and without ID/anaemia achieved intakes of 18.0 and 40.0 mg/day and 12.8 and 25.0 mg/day, respectively (Fig. 4).

## Discussion

This cross-sectional study examined the prevalence of iron supplementation during pregnancy in a group of 207 women who had recently given birth and were recruited by their midwives during regular postpartum visits. Two thirds of women (65.7%) reported to have taken iron supplements during their pregnancy. This is in accordance with results of a study carried out in Munich about ten years ago where 65.3% of women had reported to have taken iron supplements during pregnancy [25]. Similarly, in France, where iron is only prescribed to women who are at risk of ID, the use of iron tripled between the first and last trimester of pregnancy and reached a maximum of 64% towards the end of pregnancy [19]. On the other hand, results of another study conducted in Berlin in the late 1990s revealed only about 20% of iron supplementation during pregnancy [26]. These differences may be explained with different survey methodologies but also with changes over time in counseling to and practice of iron supplementation by pregnant women.

In the present study, women with a diagnosed ID/anaemia were significantly more likely to have taken an iron supplement than women with no ID/anaemia (*p* <  0.001). However, 15% of indiscriminate iron supplementation by women with no ID/anaemia or women who had not been examined had been observed. On the other hand, there was a small group of women with ID/anaemia (*n* = 7; 5.7%) who still had not used iron supplements.

Multivariate analysis revealed only one other factor, i.e. the use of other dietary supplements, independently related with iron supplementation during pregnancy. Other studies have shown that higher education [25, 28, 29] or older age [29] positively influenced iron supplementation or compliance with national iron supplementation recommendations during pregnancy. The fact that education of women in our study had no influence on the intake of iron supplements may be explained with the high proportion of study participants with higher education.

Iron supplements used by our study group contained a median dose of 80 mg/day (range: 5–200 mg/day). It is noteworthy that only about half of the women with ID/anaemia (47.5%) reported to have supplemented an iron dose of ≥80 mg/day, whereas more than one third of them had supplemented ≤40 mg/day (28.8%) or no iron at all (5.6%). The majority (58.5%) reported to have consumed an iron-only supplement. Furthermore, results revealed that nearly three quarters of women (74.1%) had supplemented iron in form of ferrous sulphate or ferrous glycine sulphate, which are most commonly used in supplements for pregnant women in Germany. While there is no evidence of different efficacies between these ferrous salts, there are many different formulations whose specific characteristics may affect the efficacy and tolerability of the product. For example, it has been suggested that some slow-release formulations release iron too far down the gastrointestinal tract for optimal absorption [30, 31]. However, one of the products used by 24.4% of the participants has been identified as best tolerated preparation with the lowest incidence of overall and gastrointestinal adverse events [32]. The WHO [33] recommends for women diagnosed with anaemia during pregnancy to supplement a dose of 120 mg/day until haemoglobin concentrations rise to normal. Against this background and in view of iron doses used by our study population, it is questionable whether women with ID/anaemia had supplemented an effective dose of iron. On the other hand, the fact that women without ID/anaemia also reported to have supplemented iron in doses of up to 80 mg/day suggests that a fraction of the study group had used unnecessarily high amounts of iron. As many of these women reported to have followed doctors’ recommendations indicates that there are medical professionals in Germany who advise routine iron supplementation in pregnancy. However, there is no information available as to why iron supplementation was recommended in these specific cases.

With regard to the onset of iron supplementation, results revealed that women who had supplemented iron because of a diagnosed ID/anaemia most often commenced supplementation in the second or third trimester of pregnancy, whereas most of those who had other reasons for taking iron supplements started in the first trimester or even before pregnancy (*p* = 0.02; see Fig. 3). This is in line with observations from other studies [19, 29, 34], and it also corresponds with the observation that most cases of ID/anaemia in pregnancy are diagnosed in the second and third trimester due to increasing needs for iron during that time [1, 35].

Although we found that women who used iron supplements were more likely to have also used other dietary supplements, the daily amount of iron consumed via these supplements was usually low (7.5–15 mg/day) and absorption of iron from normal dietary supplements has not been investigated adequately, but is also considered to be low [31]. Claims about the contribution of conventional multi-nutrient supplements to iron supply can therefore be misleading for pregnant women, especially in cases of ID/anaemia. The observation that women who reported to have consumed iron-fortified foods had a greater tendency to have used iron supplements (*p* = 0.06) suggests that pregnant women pay particular attention to their diet and try to integrate additional sources of iron into the diet. Although these self-selected foods are usually not sufficient to treat women with ID/anaemia, they should be taken into consideration when counselling non-anaemic women.

Our results indicate that the median iron intake from food, including iron-fortified foods and conventional multi-nutrient supplements, was 8 mg/day and thus more than 50% lower than the reference value of 16 mg/day set for pregnant women by EFSA [4] and far lower than the reference value of 30 mg/day recommended for pregnant women by the German Nutrition Society [5]. The intake amount calculated here was also lower than that reported from the German National Food Consumption Survey (NVS II) (median: 11.3–12.3 mg/day), which may be explained by different methodologies used to determine the intake. Noteworthy is that differences in iron exposure (excluding iron supplements, see Fig. 4) between women with and without diagnosed ID/anaemia were only seen in the upper percentiles of the study group, e.g. the 90th percentile of women with and without ID/anaemia was at 40.0 and 25 mg/day, respectively. This difference could be due to the fact that part of the women diagnosed with ID/anaemia had increasingly integrated iron-fortified foods and food supplements containing iron amongst other micronutrients into their diet. However, the observation of only small differences in iron intake between the two groups also provides evidence that iron status in pregnancy is not so much related with dietary iron intake but more with the duration and intensity of menstrual bleeding, which in turn is influenced by both genetic factors and methods of contraception [20, 35, 36].

In the present study, the prevalence of anaemia, as reported by participants, was 26.6% in the second and 19.3% in the third trimester. These figures are in agreement with data presented by WHO [24] for pregnant women in Europe (mean: 24.5%; range: 17.8 to 33.8%). They are also in accordance with data from healthy Danish pregnant women (not taking iron supplements) of whom 21% were diagnosed with IDA [31]. However, in other studies from Germany [26, 35] lower anaemia prevalence of 13.6% right before delivery and of 6% up to the 28th gestation week were observed. These differences may be explained by variations in timing of iron status examinations and presumably also by divergent threshold values applied to discriminate between anemic and non-anemic women: Maternity guidelines^2^ in Germany indicate that haemoglobin should be measured early in pregnancy and afterwards, depending on the outcome at the first examination, every four weeks or, in case of normal levels at baseline, again in the third trimester. While the German maternity guidelines recommend a cut-off for haemoglobin of 112 g/L, WHO proposes a threshold of 110 g/L while recognising that haemoglobin concentrations drops by approximately 5 g/L in the second trimester [33]. Since women in our study only reported whether or not they had been identified with ID/anaemia, nothing can be said about the threshold values applied in cases of ID/anaemia. However, following the German maternity guidelines will result in a higher prevalence of ID/anaemia than applying the WHO cut-offs. Also, iron status diagnostics in pregnancy is generally limited to measurement of haemoglobin in Germany. Although this is the most reliable indicator of anaemia at the population level, measurements of this concentration alone do not determine the cause of anaemia [33]. Therefore, further research should be undertaken to verify the prevalence of ID/anaemia, preferably not only based on measures of haemoglobin but also of other iron status biomarkers such as ferritin and soluble transferrin receptor [35].

As there are limited contemporary data on iron supplement use by pregnant women in Germany, this study presents detailed and up-to-date information on prevalence and doses of iron supplementation (and intake of iron via other food sources) of a limited sample of women who reported about their recent pregnancy as well as on commencement and duration of iron supplementation and associations with sociodemographic and maternal factors. Strengths of this study were that women from different parts of Germany participated and thus provided an overview of various counselling and supplementation practices. In this context, recruitment of participants through midwives was helpful because direct recruitment by the study team would not have been practicable and, besides, women in childbed often have a good relationship to their attending midwives, which might have had a positive effect on their willingness to participate. On the other hand, the relatively low response rate of 26.5%, which is considered a weakness and a significant source of bias of this study, can surely be explained by the recruitment process, which did not allow for direct contact between participants and the study team, but also by the fact that recruiting women in childbed is generally challenging because postnatal women are usually occupied with their newborns. It also has to be acknowledged that women in the present study were predominantly of German origin and had a higher educational level.

Further limitations of this study were that the study group was not at all representative for pregnant women in Germany; that data collection was retrospective and that information about the participants’ iron status relied on self-reported data and could not be validated. Moreover, other health parameters of the study group such as the prevalence of gestational diabetes, but also duration of gestation or birth weight of the infants were not recorded.

## Conclusion

Altogether, iron is essential for regular foetal development and growth. Dietary reference values set for iron can only be achieved by a small percentage of pregnant women through normal dietary intake. The majority of pregnant women included here supplemented iron because of a diagnosed ID/anaemia. However, not all women with ID/anaemia supplemented (sufficient amounts of) iron, while indiscriminate use of iron supplements was also observed in women without ID/anaemia. Study results, though not representative or generalizable to pregnant women in Germany, are informative and suggest that further research is warranted with regard to the validity of biomarkers and cut-offs applied for assessment of ID/anaemia in pregnancy, but also to the question of an appropriate use of iron supplements with respect to effectiveness and safety.

## Additional file

Additional file 1: Questionnaire on iron supplementation during pregnancy. To clearly present the collection of data, an English version of the questionnaire is attached as an additional file. It enables readers to carry out further studies. (PDF 457 kb)

## Abbreviations

CI: Confidence interval
D-A-CH: Nutrition Societies of Germany, Austria and Switzerland
DRV: Dietary reference value
EFSA: European Food Safety Authority
ID: Iron deficiency
IDA: Iron deficiency anaemia
NVS II: National Food Consumption Survey
OR: odds ratio
WHO: World Health Organisation

## Footnotes

1 A validated Food Frequency Questionnaire [37] was used with permission from the Robert Koch-Institute in Germany.

2 Guidelines of the Joint Federal Committee concerning medical care during pregnancy and after childbirth (“Mutterschafts-Richtlinien”) in the version of 10 December 1985 (published in the Federal Gazette No. 60a of 27 March 1986) last amended on 21 April 2016, published in the Federal Gazette AT 19.07.2016 B5 entered into force on 20 July 2016.
