# Supplementary material for: Iron supplementation during pregnancy – a cross-sectional study undertaken in four German states
Source: BMC Pregnancy Childbirth. 2018 Dec 13;18:491. doi: 10.1186/s12884-018-2130-5 (PMC6293639; doi:10.1186/s12884-018-2130-5)
Supplement: Supplementary file 1 — Questionnaire on iron supplementation during pregnancy. To clearly present the collection of data, an English version of the questionnaire is attached as an additional file. It enables readers to carry out further studies. (PDF 457 kb) [file 12884_2018_2130_MOESM1_ESM.pdf]

Code number: _____

Date of questionnaire completion: ____/____/_____

Date of delivery: ____/____/_____

In the following you'll be asked about the intake of iron via supplements during your last pregnancy. Please note that iron supplements comprise dietary supplements as well pharmaceuticals that contain iron and can be bought in pharmacies or drug stores. They are available as tablets or capsules,
but also drops or powders.

Please note that all questions about your pregnancy refer to the most recent pregnancy.

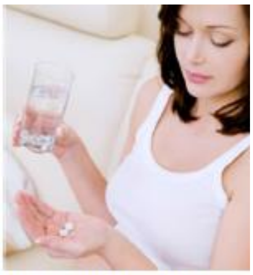
Bildquelle: shutterstock

Part A - Questions about your pregnancy

1. **Is this your first child?**
   - o Yes → *please continue with question 3*
   - o No

2. **When was your previous child born?**
   - o Year: __ __ __ __

3. **Have you ever had your iron status checked while you were pregnant with your present child?**
   - o Yes
   - o No → *please continue with question 6*

4. **If yes, what was the outcome?**
   - o Iron deficiency
   - o Parameters were within the normal range → *please continue with question 6*
   - o I don't know → *please continue with question 6*

5. **At what stage of your pregnancy were you diagnosed with iron deficiency?**
   - o In the first trimester (1st to 3rd month)
   - o In the second trimester (4th to 7th month)
   - o In the third trimester (8th to 9th/10th month)

6. **Did you take iron supplements during pregnancy?**
   - o Yes
   - o No → *please continue with question 13*

7. **Why did you take iron supplements?** (Multiple answers possible!)
    - I had iron deficiency diagnosed
    - I was advised to do so by/Because of recommendations of:
        - A doctor
        - A pharmacist
        - A midwife
        - Another person: ____
    - I follow a vegetarian diet
    - I follow a vegan diet
    - I heard that pregnant women need more iron
    - I got information from the media (television/internet/radio)
    - I read about it (magazines/brochures/books)
    - Other reasons: ____
    - No statement

8. **When did you start taking iron supplements?** (Multiple answers possible!)
    - Long before pregnancy
    - When I planned to become pregnant
    - Once I knew I was pregnant
    - Once I knew I have an iron deficiency
    - In week ____ of pregnancy
    - I don't know
    - Other answers: ____

9. **For how long did you take iron supplements?**
    - Until the end of pregnancy
    - Until my iron status was deemed normal
    - For ____ weeks
    - I don't know
    - Other answers: ____

10. **Do you know the name of the iron supplement you used?**
    (If you supplemented more than one, please refer to the supplement you took the longest.)
    - Yes, name of the supplement: ____
    - No
    - No statement

11. **How much iron did you take per day via this supplement?**
    - ____ mg
    - I don't know

12. **How often did you take the iron supplement?**
    - o Once per day
    - o Once per week
    - o Irregularly
    - o Other answers: ______________________________

13. **Did you supplement other nutrients during pregnancy?**
    - o Yes
    - o No → *please continue with question 15*

14. **What other supplements (apart from iron) did you take?** (If possible, please provide the product name/s)

    ____

    ____

Part B - Questions about your food consumption during the past four weeks

15. **What diet did follow during the past four weeks?**
    - o I followed a mixed diet (foods of animal origin and plant-based foods) → *please continue with question 17*
    - o I followed a vegetarian diet
    - o I followed a vegan diet
    - o Other answers: ______________________________

16. **If you followed a vegetarian or vegan diet, what food did you refuse to eat?**
    - o Meat, poultry, sausage
    - o Fish and seafood
    - o Milk and milk products
    - o Eggs and egg products
    - o Other answers: ______________________________

17. **Did you enquire information about the topic of nutrition during pregnancy?**
    - o Yes
    - o No → *please continue with question 19*

18. **Where did you enquire information about nutrition in this phase of life?** (Multiple answers possible!)
    - o Doctor/Pharmacists
    - o Midwife
    - o Friends/family/acquaintances
    - o Magazines/brochures/books
    - o Other answers: ______________________________

19. **Did you deliberately change your diet during pregnancy?**
    - o Yes
    - o No → *please continue with question 21*

20. **If you changed your diet, what did you change?**

    ____

    ____

Consumption of beverages during the past 4 weeks

21. **How often did you drink fruit juice (e.g. orange, apple, cherry juice)?**

    o never → *please continue with question 23*

    o 1 x per month
    o 2-3 x per month
    o 1-2 x per week
    o 3-4 x per week
    o 5-6 x per week
    o 1 x per day
    o 2 x per day
    o 3 x per day
    o 4-5 x per day
    o more than 5 x per day

22. **If you did drink fruit juice, how much did you drink on average?**

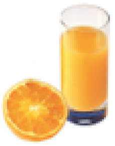

    o ½ glass (or less)
    o 1 glass (200 ml)
    o 2 glasses
    o 3 glasses
    o 4 glasses (or more)

23. **How often did you drink coffee (plus cappuccino, latte macchiato, espresso)?**

    o never → *please continue with question 25*

    o 1 x per month
    o 2-3 x per month
    o 1-2 x per week
    o 3-4 x per week
    o 5-6 x per week
    o 1 x per day
    o 2 x per day
    o 3 x per day
    o 4-5 x per day
    o more than 5 x per day

24. **If you did drink coffee, how much did you drink on average?**

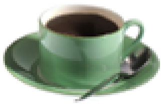

    o ½ cup (or less)
    o 1 cup (200 ml)
    o 2 cups
    o 3 cups
    o 4 cups (or more)

25. **How often did you drink black or green tea?**

- o never → *please continue with question 28*
- o 1 x per month
- o 2-3 x per month
- o 1-2 x per week
- o 3-4 x per week
- o 5-6 x per week
- o 1 x per day
- o 2 x per day
- o 3 x per day
- o 4-5 x per day
- o more than 5 x per day

26. **If you did drink black or green tea, how much did you drink on average?**

- o ½ cup (or less)
- o 1 cup (200 ml)
- o 2 cups
- o 3 cups
- o 4 cups (or more)

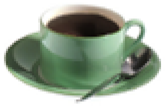

27. **Did you drink those beverages during one of the three main meals?**

Fruit juice	Tea:	Coffee:
o Yes	o Yes	o Yes
o No	o No	o No
o Sometimes	o Sometimes	o Sometimes
o Not consumed	o Not consumed	o Not consumed

Consumption of fruits and vegetables during the past 4 weeks

28. **How often did you eat (raw or cooked) vegetables?**

- o never → *please continue with question 30*
- o 1 x per month
- o 2-3 x per month
- o 1-2 x per week
- o 3-4 x per week
- o 5-6 x per week
- o 1 x per day
- o 2 x per day
- o 3 x per day
- o 4-5 x per day
- o more than 5 x per day

29. **If you did eat vegetables, how much did you eat on average per meal?**

- o ¼ portion (or less)
- o ½ portion
- o 1 portion (see figure)
- o 2 portions
- o 3 portions (or more)

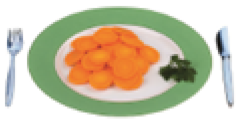

30. **How often did you eat (fresh or cooked) fruits?**

- o never → *please continue with question 33*
- o 1 x per month
- o 2-3 x per month
- o 1-2 x per week
- o 3-4 x per week
- o 5-6 x per week
- o 1 x per day
- o 2 x per day
- o 3 x per day
- o 4-5 x per day
- o more than 5 x per day

31. **If you did eat fruits, how much did you eat on average per meal?**

- o ½ portion (or less)
- o 1 portion (see figure)
- o 2 portions
- o 3 portions
- o 4 portions (or more)

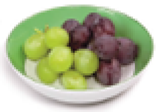

32. **Did you eat fruits during the main meals?**

- o Yes
- o No, but directly after the main meals (as a desert)
- o No, but as a snack between meals
- o Time of consumption varied

33. **How often did you eat pulses (e.g. beans, peas, lentils)?**

- o never → *please continue with question 35*
- o 1 x per month
- o 2-3 x per month
- o 1-2 x per week
- o 3-4 x per week
- o 5-6 x per week
- o 1 x per day
- o 2 x per day
- o 3 x per day
- o 4-5 x per day
- o more than 5 x per day

34. **If you did eat pulses, how much did you eat on average per meal?**

- o ¼ portion (or less)
- o ½ portion
- o 1 portion (see figure)
- o 2 portions
- o 3 portions (or more)

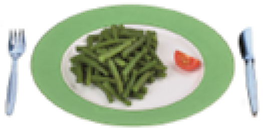

Consumption of meat and sausages during the past 4 weeks

35. **How often did you eat meat (except sausages)? (e.g. chicken, beef, pork, game meat)**

- o never → *please continue with question 37*
- o 1 x per month
- o 2-3 x per month
- o 1-2 x per week
- o 3-4 x per week
- o 5-6 x per week
- o 1 x per day
- o 2 x per day
- o 3 x per day
- o 4-5 x per day
- o more than 5 x per day

36. **If you did eat meat, how much did you eat on average per meal?**

- o ¼ portion (or less)
- o ½ portion
- o 1 portion (see figures)
- o 2 portions
- o 3 portions (or more)

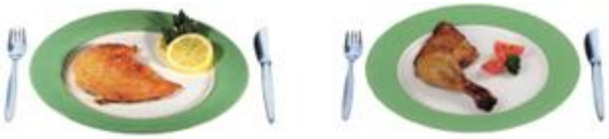

37. **How often did you eat sausages (e.g. liver sausage, ham)?**

- o never → *please continue with question 39*
- o 1 x per month
- o 2-3 x per month
- o 1-2 x per week
- o 3-4 x per week
- o 5-6 x per week
- o 1 x per day
- o 2 x per day
- o 3 x per day
- o 4-5 x per day
- o more than 5 x per day

38. **If you did eat sausages, how much did you eat on average per meal?**

- o ½ slice
- o 1 slice
- o 2 slices (see figure)
- o 3 slices
- o 4 slices (or more)

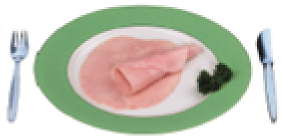

Consumption of fish during the past 4 weeks

39. **How often did you eat fish as a cold or warm meal (e.g. smoked salmon, pickled herring, tuna, saithe, trout)**

- o never → *please continue with question 41*
- o 1 x per month
- o 2-3 x per month
- o 1-2 x per week
- o 3-4 x per week
- o 5-6 x per week
- o 1 x per day
- o 2 x per day
- o 3 x per day
- o 4-5 x per day
- o more than 5 x per day

40. **If you did eat fish, how much did you eat on average per meal? (One portion is equivalent to 1 fish fillet, 4 fish fingers or the amount of a topping.)**

- o ¼ portion (or less)
- o ½ portion
- o 1 portion
- o 2 portions
- o 3 portions (or more)

Consumption of grain products during the past 4 weeks

41. **How often did you eat cereals?**

- o never → *please continue with question 43*
- o 1 x per month
- o 2-3 x per month
- o 1-2 x per week
- o 3-4 x per week
- o 5-6 x per week
- o 1 x per day
- o 2 x per day
- o 3 x per day
- o 4-5 x per day
- o more than 5 x per day

42. **If you did eat cereals, how much did you eat on average per meal?**

- o ¼ bowl (or less)
- o ½ bowl
- o 1 bowl (see figure, 150 ml)
- o 2 bowls
- o 3 bowls (or more)

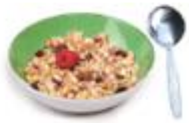

43. **How often did you eat wholemeal bread or wholemeal buns?**

- o never → *please continue with question 45*
- o 1 x per month
- o 2-3 x per month
- o 1-2 x per week
- o 3-4 x per week
- o 5-6 x per week
- o 1 x per day
- o 2 x per day
- o 3 x per day
- o 4-5 x per day
- o more than 5 x per day

44. **If you did eat wholemeal bread or wholemeal buns, how much did you eat on average per meal?**

- o ½ slice or ½ bun (or less)
- o 1 slice or ½ bun
- o 2 slices or 2 buns
- o 3 slices or 3 buns
- o 4 slices or 4 buns (or more)

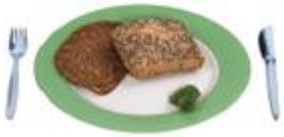

45. **How often did you eat brown rice?**

- o never → *please continue with question 47*
- o 1 x per month
- o 2-3 x per month
- o 1-2 x per week
- o 3-4 x per week
- o 5-6 x per week
- o 1 x per day
- o 2 x per day
- o 3 x per day
- o 4-5 x per day
- o more than 5 x per day

46. **If you did eat brown rice, how much did you eat on average per meal?**

- o ¼ portion (or less)
- o ½ portion
- o 1 portion (see figure)
- o 2 portions
- o 3 portions (or more)

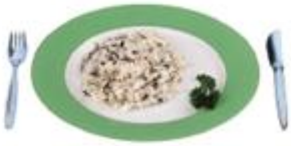

47. **How often did you eat wholemeal noodles?**

- o never → *please continue with question 49*
- o 1 x per month
- o 2-3 x per month
- o 1-2 x per week
- o 3-4 x per week
- o 5-6 x per week
- o 1 x per day
- o 2 x per day
- o 3 x per day
- o 4-5 x per day
- o more than 5 x per day

48. **If you did eat wholemeal noodles, how much did you eat on average per meal?**

- o ¼ portion (or less)
- o ½ portion
- o 1 portion (see figure)
- o 2 portions
- o 3 portions (or more)

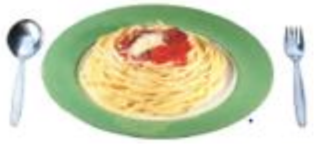

49. **How often did you eat eggs (e.g. fried egg, scrambled egg, boiled egg)?**

- o never → *please continue with question 51*
- o 1 x per month
- o 2-3 x per month
- o 1-2 x per week
- o 3-4 x per week
- o 5-6 x per week
- o 1 x per day
- o 2 x per day
- o 3 x per day
- o 4-5 x per day
- o more than 5 x per day

50. **If you did eat eggs, how much did you eat on average per meal?**

- o ½ egg
- o 1 egg
- o 2 eggs
- o 3 eggs
- o 4 eggs (or more)

51. **Did you drink juices enriched with iron during the past four weeks?**

- ○ No
- ○ Yes → please provide product name, amount and frequency:

Product name: ____________________________________

_____ glass/glasses per ______[day/week/month]

52. **Did you eat cereals enriched with iron during the past four weeks?**

- ○ No
- ○ Yes → please provide product name, amount and frequency:

Product name: ____________________________________

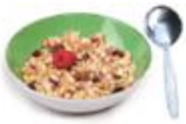

_____ portion/portions per ______[day/week/month]

53. **Did you eat or drink any other foods marketed specifically for pregnant women during the past four weeks?**

- ○ No
- ○ Yes, product name: ____

54. **Did you smoke cigarettes during pregnancy?**

- ○ No, I generally don’t smoke
- ○ Once I knew I was pregnant, I stopped smoking
- ○ I did smoke during pregnancy: Amount per week: ______ cigarette/cigarettes
- ○ No statement

55. **Did you drink alcohol during pregnancy?**
   - o No, I generally don’t drink alcohol
   - o Once I knew I was pregnant, I stopped drinking alcohol
   - o I did drink alcohol during pregnancy: Amount per week: ______ glass/glasses
   - o No statement

Part C – Personal data

56. **How old are you?**
__ __ years

57. **What is your country of birth?**
   - o Germany → *please continue with question 59*
   - o Other country: ____________________

58. **If you were born outside Germany, for how long have you been living in Germany?**
Since [year]: __ __ __ __

59. **What is the highest degree or level of school you have completed?**
   - o Basic secondary school
   - o Intermediate secondary school
   - o Abitur (A level)
   - o Vocational school/ technical college
   - o University/ University of Applied Science

60. **Where do you live?** (Please provide the **postal code**!)
— — — — —

Thank you for your participation in the study!
